# Prevalence and correlates of experiencing drug-related discrimination among people who use drugs presenting at emergency department at high risk of opioid overdose

**DOI:** 10.1016/j.abrep.2023.100496

**Published:** 2023-05-18

**Authors:** Shayla Nolen, Taneisha Wilson, Brendan P. Jacka, Yu Li, Francesca L. Beaudoin, Brandon D.L. Marshall

**Affiliations:** a Department of Epidemiology, Brown University School of Public Health, 121 South Main St, Box G-S-121-2, Providence, RI 02912, USA; b Department of Emergency Medicine, Warren Alpert Medical School of Brown University, Providence, RI 02903, USA

## Abstract

**Objectives:**

Our objective is to determine if specific sociodemographic characteristics were associated with perceived drug-related discrimination among people who use drugs (PWUD) presenting for care in the emergency department (ED).

**Methods:**

We conducted a secondary analysis of data from the Navigator trial, a randomized control trial of two behavioral interventions in the ED for people at risk of an opioid overdose. Participants included adult patients presenting to two Rhode Island EDs. Eligible participants included those high risk for an opioid overdose, resided or received most of their healthcare in Rhode Island, and were able to provide consent. The primary outcome of this analysis was self-reported feelings of drug-related discrimination by the medical community. The independent variables of interest included race/ethnicity, gender identity, and sexual orientation. Log-binomial multivariable regression models were constructed with all three independent variables of interest and a selection of sociodemographic covariates.

**Results:**

Of 620 eligible participants, 251 (40.5%) reported ever experiencing drug-related discrimination in their lifetime. In the adjusted model, participants who identified as women and participants who identified as LGBQIA+ were more likely to report experiencing drug-related discrimination from the medical community in EDs. Racial/ethnic minority groups were less likely than White (non-Hispanic) participants to report drug-related discrimination.

**Discussion:**

In this study population, White participants reported more drug-related discrimination than their minority counterparts, although female and LGBQIA+ patients reported more discrimination. Future studies should further assess the significance of these intersecting identities on self-reported discrimination. This knowledge could improve ED-based interventions, policies, and services for PWUD.

## Highlights

• 40.5% of PWUD reported drug-related discrimination in emergency departments.
• PWUD who identified as women were more likely to report drug-related discrimination.
• PWUD who identified as LGBQIA+ were also more likely to report discrimination in EDs.

## 1 Introduction

Despite an elevated risk of adverse health outcomes (e.g., chronic diseases, infections), people who use drugs (PWUD) are more likely to delay seeking medical treatment because of concerns of drug-related stigma and discrimination by the medical community (Meyerson et al., 2021, Muncan et al., 2020, McKnight et al., 2017, Biancarelli et al., 2019). Within the PWUD population, additional discrimination related to classism, racism, sexism, and homophobia may exacerbate the consequences of drug-related stigma (Acevedo et al., 2018, Collins et al., 2019, Hagle et al., 2021, Jacobson et al., 2007, Kosteniuk et al., 2022, Matsuzaka and Knapp, 2020, McNeil et al., 2016, Mendoza et al., 2019). PWUD who are racial/ethnic minorities, are part of the LGBTQ+ community, and those experiencing unstable housing are less likely to enter substance use disorder treatment programs and have poorer access to harm reduction services, which leads to health concerns becoming more severe than their counterparts who did not use drugs (Hagle et al., 2021, Matsuzaka and Knapp, 2020, Cochran and Cauce, 2004, Flentje et al., 2015, Flentje et al., 2015, Jeopardy, 2020:).

One of the healthcare settings in which PWUD commonly report experiencing drug-related discrimination is the emergency department (ED) (Muncan et al., 2020, Kattari et al., 2015). With many PWUDs using the ED as their primary source of healthcare, the ED is an opportune place to intervene to provide treatment referrals, recovery support services, and harm reduction supplies (Doupe et al., 2012, Moulin et al., 2018). However, PWUD have perceived that care has been withheld by providers in the ED because of their drug use status. (Volkow, 2020) Additional research is needed to understand the role of stigma and discrimination in creating barriers to harm reduction services and treatment engagement in the ED. In addition, it is unknown if the effect is differential across various subpopulations (e.g., racial/ethnic minorities, women and people who part of the LGBTQ+ community). Therefore, we aimed to determine the relationship between sociodemographic characteristics on perceived drug-related discrimination among PWUD in the ED setting.

## 2 Methods

This study is a secondary analysis of data from the Navigator trial, a randomized control trial of two behavioral interventions in the ED for people at risk of an opioid overdose, which has previously been described in detail. (Goedel et al., 2019, Beaudoin et al., 2022) Briefly, adults presenting at two EDs in Rhode Island from November 2017–May 2021 were eligible if they were at high risk for an opioid overdose event (i.e., the current visit was for an opioid overdose, received treatment related to opioid use disorder at the time of visit, or reported an opioid overdose within the previous twelve months), resided and/or received most of their healthcare in Rhode Island, and provided consent to participate. Individuals who were pregnant or under law enforcement supervision were not eligible. At the time of enrollment, enrolled participants completed a comprehensive behavioral survey at the time of the ED visit and were compensated $40 for their time. This parent study was reviewed and approved by Rhode Island/Miriam Hospital Institutional Review Board.

The primary outcome for this analysis was reporting ever feeling discriminated against by the medical community (e.g., a doctor, nurse, or clinic staff) due to their current or past drug use. The independent variables of interest included race/ethnicity, current gender identity, and sexual orientation. Self-reported race and ethnicity responses were then categorized into four mutually exclusive groups: African American/Black (non-Hispanic), Hispanic (any race), Other (non-Hispanic), and White (non-Hispanic). The Other category included American Indian/Alaska Native, Asian, Native Hawaiian/Pacific Islander, Mixed/Bi-racial/Multi-racial, and Self-Defined. Participants were asked what best described their current gender identity. Gender identities were categorized into three groups to minimize small cell sizes: woman, man, and non-binary/transgender/other. Lastly, sexual orientation was collected and categorized into two groups, straight and LGBQIA+.

Individual bivariable analyses and log-binomial regression models were conducted for each independent variable of interest and the following covariates: age, highest level of education (less than high school, high school or GED, some college, and college/university degree or higher), having children under the age of 17 years old (yes vs. no), pre-pandemic enrollment (i.e., March 1, 2020, yes vs. no), having health insurance (yes vs. no), previous experience with substance use disorder treatment (yes vs. no), ever receiving professional or non-professional recovery services (yes vs. no), ever tried to enroll into treatment but was unable to (yes vs. no), ever incarcerated (yes vs. no), ever homeless (yes vs. no), currently employed full time (yes vs. no), and average monthly income ($0, $1 - $500, $501 - $1500, $1501 - $3000, and >$3000). Next, we constructed multivariable regression models with all three independent variables of interest and the following covariates: previous experience with substance use disorder treatment, ever incarcerated and ever homeless. All covariates included in the model were systematically assessed for inclusion into the final model using backward elimination approach. Participants were excluded from the analysis if they were missing the independent or primary dependent variables; missing covariate data were recorded as unknown. Analyses were conducted using R version 3.5.2 and SAS version 9.4 (SAS Institute, Cary, North Carolina).

## 3 Results

Of the 648 participants included in the parent trial, 28 (4.0%) participants were excluded for having missing data on race/ethnicity, gender, sexual orientation, or the study outcome. There were no statistically significant differences in demographics between those who were excluded from the study and those who remained.The remaining 620 eligible participants were predominantly White (non-Hispanic) (66.9%), identified as a man (68.1%), and straight (88.9%, see Table 1). A total of 251 (40.5%) reported ever experiencing drug-related discrimination in their lifetime.

**Table 1:** Descriptive characteristics of those who experienced discrimination by the medical community due to current or previous drug use, N = 620.

	Overall (n = 620) N (%)	Did Not Experience Discrimination (n = 369) N (%)	Experienced discrimination (n = 251) N (%)	p-value
Race/Ethnicity				<0.001
African American/Black (non-Hispanic)	45 (7.3)	32 (8.6)	13 (5.1)	
Hispanic (any race)	107 (17.3)	79 (21.4)	28 (11.0)	
Other (non-Hispanic)	53 (8.5)	35 (9.5)	18 (7.2)	
White (non-Hispanic)	415 (66.9)	223 (60.4)	192 (76.5)	
Age, Mean (SD)	36.9 (10.8)	37.1 (11.3)	36.6 (10.0)	0.521
Gender				0.006
Women	197 (31.8)	101 (27.4)	96 (38.2)	
Men	422 (68.1)	268 (72.6)	155 (61.0)	
Non-binary/Transgender	1 (0.1)	0 (0.0)	1 (0.4)	
Sexual Orientation				<0.001
LGBQIA+	69 (11.1)	27 (7.3)	42 (16.7)	
Straight	551 (88.9)	342 (92.2)	209 (83.3)	
Highest Level of Education				<0.001
Less than High School	180 (29.0)	119 (32.2)	61 (24.3)	
High School or GED	223 (36.0)	147 (39.8)	76 (30.3)	
Some College or Trade School	164 (26.5)	76 (20.6)	88 (35.1)	
College/University Degree or Higher	51 (8.2)	25 (6.8)	26 (10.4)	
Don’t Know/Refused to Answer	2 (0.3)	2 (0.5)	0 (0.0)	
Children Under the Age of 17 Years Old				0.214
Yes	94 (15.2)	50 (13.6)	44 (17.5)	
No	526 (84.8)	319 (86.4)	207 (82.5)	
Don’t Know/Refused to Answer	0 (0.0)	0 (0.0)	0 (0.0)	
Enrolled During COVID-19 Pandemic				0.145
Yes	266 (42.9)	149 (40.4)	117 (46.6)	
No	354 (57.1)	220 (59.6	134 (53.4)	
Don’t Know/Refused to Answer	0 (0.0)	0 (0.0)	0 (0.0)	
Have Health Insurance				0.479
Yes	556 (78.4)	333 (90.2)	223 (88.8)	
No	48 (20.2)	27 (7.3)	21 (8.4)	
Don’t Know/Refused to Answer	16 (2.5)	27 (7.3)	7 (2.8)	
Have Been in Treatment Before				<0.001
Yes	486 (78.4)	264 (71.5)	222 (88.4)	
No	125 (20.2)	100 (27.1)	25 (10.0)	
Don’t Know/Refused to Answer	9 (1.4)	5 (1.4)	4 (1.6)	
Ever Received any Recovery Services				0.002
Yes	215 (34.7)	110 (29.8)	105 (41.8)	
No	401 (64.7)	258 (69.9)	143 (57.0)	
Don’t Know/Refused to Answer	4 (0.7)	1 (0.3)	3 (1.2)	
Ever Tried to Enroll in Treatment but Was Unable to				<0.001
Yes	217 (35.0)	273 (74.0)	125 (49.8)	
No	392 (63.2)	92 (24.9)	119 (47.4)	
Don’t Know/Refused to Answer	11 (1.8)	4 (1.1)	7 (2.8)	
Ever Incarcerated				0.033
Yes	433 (69.3)	242 (65.6)	187 (74.5)	
No	92 (14.7)	56 (15.2)	36 (14.3)	
Don’t Know/Refused to Answer	100 (16.0)	71 (19.2)	28 (11.2)	
Ever Homeless				<0.001
Yes	432 (69.7)	235 (63.7)	197 (78.5)	
No	186 (30.0)	133 (36.0)	53 (21.1)	
Don’t Know/Refused to Answer	2 (0.3)	1 (0.3)	1 (0.4)	
Currently Employed				0.164
Yes	175 (28.2)	115 (31.2)	60 (23.9)	
No	437 (70.5)	248 (67.2)	189 (75.3)	
Don’t Know/Refused to Answer	8 (1.3)	6 (1.6)	2 (0.8)	
Average Monthly Income				0.479
$0	153 (24.7)	85 (23.0)	68 (27.1)	
$1 - $500	86 (13.9)	46 (12.5)	40 (15.9)	
$501 - $1500	189 (30.5)	118 (32.0)	71 (28.3)	
$1501 - $3000	95 (15.3)	62 (16.8)	33 (13.1)	
>$3000	53 (8.5)	29 (7.9)	24 (9.6)	
Don’t Know/Refused to Answer	41 (6.6)	27 (7.3)	14 (5.6)	

The unadjusted log-binomial models found African American/Black (non-Hispanic) (Prevalence Ratio [PR]: 0.62, 95% Confidence Interval [CI]: 0.37, 0.95) and Hispanic (PR: 0.57, 95% CI: 0.39, 0.77) participants had a lower prevalence of reporting experiencing discrimination by the medical community for their current or previous drug use compared to White (non-Hispanic) participants (Table 2). In contrast, the prevalence for participants who identified as women was 1.34 times higher than men (PR: 1.34, 95% CI: 1.10, 1.61), and participants who identified as LGBQIA+ had a 60% higher prevalence of reporting experiencing discrimination in comparison to participants who identified as straight (PR: 1.60, 95% CI: 1.27, 1.96). In the adjusted model, participants who identified as women (PR: 1.26, 95% CI: 1.05, 1.52) and participants who identified as LGBQIA+ (PR: 1.54, 95% CI: 1.28, 1.86) had a higher prevalence of reporting experiencing discrimination from the medical community in comparison to their counterparts. Other covariates associated with experiencing drug-related discrimination are shown in Table 2.

**Table 2:** Log-binomial Regression of those who experienced discrimination by the medical community due to current or previous drug use.

	Unadjusted PR (95% CI)	Adjusted PR (95% CI)
Race/Ethnicity		
African American/Black (non-Hispanic)	**0.62 (0.37, 0.95)**	0.69 (0.42, 1.12)
Hispanic (any race)	**0.57 (0.39, 0.77)**	0.79 (0.56, 1.12)
Other (non-Hispanic)	0.73 (0.47, 1.04)	0.70 (0.47, 1.04)
White (non-Hispanic)	ref	ref
Age, Mean (SD)	1.00 (0.99, 1.01)	–
Gender		
Women	**1.34 (1.10, 1.61)**	**1.26 (1.05, 1.52)**
Men	ref	ref
Sexual Orientation		
LGBQIA+	**1.60 (1.27, 1.96)**	**1.54 (1.28, 1.86)**
Straight	ref	ref
Highest Level of Education		
Less than High School	ref	–
High School or GED	1.01 (0.53, 1.33)	–
Some College	**1.58 (1.24, 2.05)**	–
College/University Degree or Higher	**1.50 (1.05, 2.08)**	–
Children Under the Age of 17 Years Old		
Yes	1.18 (0.92, 1.48)	–
No	ref	–
Enrolled During COVID-19 Pandemic		
Yes	1.16 (0.96, 1.41)	–
No	ref	–
Have Health Insurance		
Yes	0.92 (0.68, 1.34)	–
No	ref	–
Ever Enrolled in Treatment		
Yes	**2.29 (1.59, 3.30)**	**2.05 (1.34, 3.14)**
No	ref	ref
Ever Received any Recovery Services		
Yes	1.37 (1.14, 1.66)	–
No	ref	–
Ever Tried to Enroll in Treatment but Was Unable to		
Yes	1.89 (1.56, 2.28)	–
No	ref	–
Ever Incarcerated		
Yes	1.15 (0.84, 1.46)	1.04 (0.80, 1.36)
No	ref	ref
Ever Homeless		
Yes	**1.60 (1.24, 2.05)**	**1.25 (0.94, 1.73)**
No	ref	ref
Currently Employed		
Yes	**0.79 (0.63, 0.99)**	–
No	ref	–
Average Monthly Income		
$0	ref	–
$1 - $500	1.04 (0.78, 1.39)	–
$501 - $1500	0.84 (0.65, 1.08)	–
$1501 - $3000	0.78 (0.56, 1.08)	–
>$3000	1.01 (0.72, 1.43)	–
Bold represent p-value < 0.05		

## 4 Discussion

In this study, EP patients at high risk for opioid overdose, experiences of drug-use related discrimination were common (40.5%), especially among participants who identified as women or LGBQIA+. Participants who identified as Hispanic (any race) or African American/Black (non-Hispanic) were less likely to report experiencing discrimination compared to their White counterparts. These findings could be a result of racial/ethnic minorities attributing the discrimination that they face in medical settings to their race/ethnicity instead of their experience using drugs.

This prevalence of participants experiencing any kind of medical discrimination in our sample of PWUD, is approximately double that reported in other studies of non-PWUD (Stepanikova and Oates, 2017, Mays et al., 2017, Hausmann et al., 2008, Nong et al., 2020). However, compared to studies that only included PWUD, our results were either similar or lower, with one study reporting a lifetime prevalence of 72% (Meyerson et al., 2021, McKnight et al., 2017, Skosireva et al., 2014). The high prevalence of medical discrimination experienced by PWUD underscores the need to address bias and stigma in the medical community in order to improve the patient experience and related health outcomes. Potential interventions for ED medical staff might target implicit bias and cultural competency (Hassen et al., 2021, Kruse et al., 2022). Additional operational and policy changes can help sustain these practices and also standardize care (Varcoe et al., 2022). Organizational and policy-level modifications that have been adopted in some settings include identifying and improving culturally unsafe systems and developing a strategic leadership committee that focuses on implementing and monitoring plans for reducing patient discrimination and holding healthcare providers accountable (Hassen et al., 2021).

Women and LGBQIA + patients were more likely to report experiencing drug-related discrimination from the medical community. These findings are corroborated by prior literature that found women and sexually diverse populations were more likely to report experiencing discrimination in healthcare settings (Hagle et al., 2021, Mendoza et al., 2019, Cochran and Cauce, 2004). Efforts to address drug-related discrimination from medical providers should incorporate the needs of gender and sexually diverse patient populations. Healthcare providers, especially those in the EDs, can improve provider-patient relationships by incorporating LGBQIA + and transgender/nonbinary health into medical and nursing school curricula, engaging with LGBQIA + organizations in the development and delivery of training, and providing gender-affirming care to patients (Allison et al., 2021).

In contrast to prior reports, racial/ethnic minorities in this sample were less likely to report experiencing discrimination due to their current or past drug use (Hagle et al., 2021, Mendoza et al., 2019, Cochran and Cauce, 2004). These disparate findings could be related to a variety of reasons, including differences in the study setting and the type of discrimination participants were asked to report (e.g., drug-related discrimination instead of racial/ethnic discrimination). All participants included in the analysis were patients presenting for care at the ED and were at high risk for an overdose. Some communities, such as racial/ethnic minorities, may be less likely to present at a hospital due to prior negative experiences (Matsuzaka and Knapp, 2020, Flentje et al., 2015). Because of this, it is possible that a subset of persons from these communities who had experienced drug-related discrimination are not present in our sample. If this is true, our sample is not necessarily generalizable to racial/ethnic minorities who are at risk for overdose. Despite these findings, more work is needed to address the negative relationship that the medical community has with racial/ethnic minorities, especially those who are PWUD. We recommend that ED leadership implement policies and programs to strengthen community relationships, increase community participation in decision-making for developing anti-racism policies, and recruit and retain racial/ethnic minorities in positions of power at all levels in the healthcare workforce (Hassen et al., 2021).

There are several limitations in our study which should be noted. First, participants included in the analysis were patients presenting at two EDs in a northeastern state of the US with a high incidence of opioid overdose, potentially limiting generalizability. Also, our survey instrument did not capture other forms of discrimination experienced by participants; therefore, we cannot determine if minority participants who did not report experiencing drug use-related discrimination were experiencing other forms of discrimination due to sex, gender, race/ethnicity, class, etc. Due to the intersectionality of participants’ identity as a racial/ethnic minority and drug use status, there are a sub-group of participants who were/are potentially experiencing discrimination, but who are attributing it to their race/ethnicity instead of their drug use (Hatcher et al., 2018). For this reason, drug-related discrimination may be underestimated in these groups. In future research on discrimination in the ED setting, other types of discrimination related to racism, sexism, homophobia, and transphobia should be included in surveys to evaluate what participants believe to be the underlying cause of the discrimination they are facing.

Although experiences of discrimination varied within patient sub-groups, the prevalence across the whole sample was quite high. This highlights the need to improve patient-provider relationships among PWUD within minority populations and integrating socially amenable policies into EDs in order to protect PWUD who experience drug-related discrimination. Additional research is needed to determine how patient identities intersect with substance use status to influence an individual’s likelihood of reporting experiencing discrimination when interacting with the medical community. This is a necessary next step for achieving the goal of improving healthcare for PWUD and implementing more effective ED-based preventative interventions for reducing overdose events.

Funding Sources:

The Navigator trial was funded by Arnold Ventures and the Cigna Foundation through investigator-initiated trial programs. The funders had no role in the design and conduct of the study; collection, management, analysis, and interpretation of the data; preparation, review, or approval of the manuscript; and decision to submit the manuscript for publication.

## CRediT authorship contribution statement

**Shayla Nolen:** Investigation, Conceptualization, Methodology, Software, Formal analysis, Writing – original draft, Writing – review & editing, Visualization. **Taneisha Wilson:** Conceptualization, Methodology, Writing – review & editing. **Brendan P. Jacka:** Conceptualization, Methodology, Writing – review & editing. **Yu Li:** Resources, Methodology, Writing – review & editing. **Francesca L. Beaudoin:** Funding acquisition, Conceptualization, Methodology, Writing – review & editing. **Brandon D.L. Marshall:** Funding acquisition, Supervision, Writing – review & editing.

## Declaration of Competing Interest

The authors declare that they have no known competing financial interests or personal relationships that could have appeared to influence the work reported in this paper.

## Data Availability

Data will be made available on request.

## References

[b0005] Meyerson B.E., Russell D.M., Kichler M., Atkin T., Fox G., Coles H.B. (2021). I don’t even want to go to the doctor when I get sick now: healthcare experiences and discrimination reported by people who use drugs, Arizona 2019. The International Journal on Drug Policy.

[b0010] Muncan B., Walters S.M., Ezell J., Ompad D.C. (2020). “They look at us like junkies”: influences of drug use stigma on the healthcare engagement of people who inject drugs in New York City. Harm Reduction Journal.

[b0015] McKnight C., Shumway M., Masson C.L. (2017). Perceived discrimination among racial and ethnic minority drug users and the association with health care utilization. Journal of Ethnicity in Substance Abuse.

[b0020] Biancarelli D.L., Biello K.B., Childs E. (2019). Strategies used by people who inject drugs to avoid stigma in healthcare settings. Drug and Alcohol Dependence.

[b0025] Acevedo A., Panas L., Garnick D. (2018). Disparities in the treatment of substance use disorders: Does where you live matter?. The Journal of Behavioral Health Services & Research.

[b0030] Collins A.B., Boyd J., Cooper H.L.F., McNeil R. (2019). The intersectional risk environment of people who use drugs. Social Science and Medicine.

[b0035] Hagle H.N., Martin M., Winograd R. (2021). Dismantling racism against Black, Indigenous, and people of color across the substance use continuum: A position statement of the association for multidisciplinary education and research in substance use and addiction. Subst Abuse..

[b0040] Jacobson J.O., Robinson P.L., Bluthenthal R.N. (2007). Racial disparities in completion rates from publicly funded alcohol treatment: Economic resources explain more than demographics and addiction severity. Health Services Research.

[b0045] Kosteniuk B., Salvalaggio G., Wild T.C., Gelberg L., Hyshka E. (2022). Perceived unmet substance use and mental health care needs of acute care patients who use drugs: A cross-sectional analysis using the Behavioral Model for Vulnerable Populations. Drug and Alcohol Review.

[b0050] Matsuzaka S., Knapp M. (2020). Anti-racism and substance use treatment: Addiction does not discriminate, but do we?. Journal of Ethnicity in Substance Abuse.

[b0055] McNeil R., Kerr T., Pauly B., Wood E., Small W. (2016). Advancing patient-centered care for structurally vulnerable drug-using populations: A qualitative study of the perspectives of people who use drugs regarding the potential integration of harm reduction interventions into hospitals. Addiction.

[b0060] Mendoza S, Hatcher AE, Hansen H. Race, Stigma, and Addiction. In: Avery JD, Avery JJ, eds. *The Stigma of Addiction: An Essential Guide*. Springer International Publishing; 2019:131-152. doi:10.1007/978-3-030-02580-9_8.

[b0065] Cochran B.N., Cauce A.M. (2004). Characteristics of lesbian, gay, bisexual, and transgender individuals entering substance abuse treatment - ScienceDirect. Journal of Substance Abuse Treatment.

[b0070] Flentje A., Heck N.C., Sorensen J.L. (2015). Substance use among lesbian, gay, and bisexual clients entering substance abuse treatment: Comparisons to heterosexual clients. Journal of Consulting and Clinical Psychology.

[b0075] Jeopardy D. (2020:). COVID-19 and behavioral health disparities for black and latino communities in the U.S. Substance Abuse and Mental Health Services Administration.

[b0080] Kattari S.K., Walls N.E., Whitfield D.L., Langenderfer-Magruder L. (2015). Racial and ethnic differences in experiences of discrimination in accessing health services among transgender people in the United States. Int J Transgenderism..

[b0085] Doupe M.B., Palatnick W., Day S. (2012). Frequent users of emergency departments: developing standard definitions and defining prominent risk factors. Annals of Emergency Medicine.

[b0090] Moulin A., Evans E.J., Xing G., Melnikow J. (2018). Substance use, homelessness, mental illness and medicaid coverage: A set-up for high emergency department utilization. The Western Journal of Emergency Medicine.

[b0095] Volkow N.D. (2020). Stigma and the toll of addiction. The New England Journal of Medicine.

[b0100] Goedel W.C., Marshall B.D.L., Samuels E.A. (2019). Randomised clinical trial of an emergency department-based peer recovery support intervention to increase treatment uptake and reduce recurrent overdose among individuals at high risk for opioid overdose: Study protocol for the navigator trial. BMJ Open.

[b0105] Beaudoin F.L., Jacka B.P., Li Y. (2022). Effect of a peer-led behavioral intervention for emergency department patients at high risk of fatal opioid overdose. JAMA Network Open.

[b0110] Stepanikova I., Oates G.R. (2017). Perceived discrimination and privilege in health care: The role of socioeconomic status and race. American Journal of Preventive Medicine.

[b0115] Mays V.M., Jones A., Delany-Brumsey A., Coles C., Cochran S.D. (2017). Perceived discrimination in healthcare and mental health/substance abuse treatment among blacks, latinos, and whites. Medical Care.

[b0120] Hausmann L.R.M., Jeong K., Bost J.E., Ibrahim S.A. (2008). Perceived discrimination in health care and health status in a racially diverse sample. Medical Care.

[b0125] Nong P., Raj M., Creary M., Kardia S.L.R., Platt J.E. (2020). Patient-reported experiences of discrimination in the US health care system. JAMA Network Open.

[b0130] Skosireva A., O’Campo P., Zerger S., Chambers C., Gapka S., Stergiopoulos V. (2014). Different faces of discrimination: Perceived discrimination among homeless adults with mental illness in healthcare settings. BMC Health Services Research.

[b0135] Hassen N., Lofters A., Michael S., Mall A., Pinto A.D., Rackal J. (2021). Implementing anti-racism interventions in healthcare settings: A scoping review. International Journal of Environmental Research and Public Health.

[b0140] Kruse J.A., Collins J.L., Vugrin M. (2022). Educational strategies used to improve the knowledge, skills, and attitudes of health care students and providers regarding implicit bias: An integrative review of the literature. International Journal of Advanced Nursing Studies.

[b0145] Varcoe C., Browne A.J., Perrin N. (2022). EQUIP emergency: Can interventions to reduce racism, discrimination and stigma in EDs improve outcomes?. BMC Health Services Research.

[b0150] Allison M.K., Marshall S.A., Stewart G., Joiner M., Nash C., Stewart M.K. (2021). Experiences of transgender and gender nonbinary patients in the emergency department and recommendations for health care policy, education, and practice. The Journal of Emergency Medicine.

[b0155] Hatcher A.E., Mendoza S., Hansen H. (2018). At the expense of a life: race, class, and the meaning of buprenorphine in pharmaceuticalized “care”. Substance Use & Misuse.

